# Sodium channel-inhibiting drugs and survival of breast, colon and prostate cancer: a population-based study

**DOI:** 10.1038/srep16758

**Published:** 2015-11-18

**Authors:** Caroline Fairhurst, Ian Watt, Fabiola Martin, Martin Bland, William J. Brackenbury

**Affiliations:** 1 Department of Health Sciences, University of York, York, UK, YO10 5DD; 2 Hull York Medical School, York, UK, YO10 5DD; 3 Department of Biology, University of York, York, UK, YO10 5DD

## Abstract

Metastasis is the leading cause of cancer-related deaths. Voltage-gated sodium channels (VGSCs) regulate invasion and metastasis. Several VGSC-inhibiting drugs reduce metastasis in murine cancer models. We aimed to test the hypothesis that patients taking VGSC-inhibiting drugs who developed cancer live longer than those not taking these drugs. A cohort study was performed on primary care data from the QResearch database, including patients with breast, bowel or prostate cancer. Cox proportional hazards regression was used to compare the survival from cancer diagnosis of patients taking VGSC-inhibiting drugs with those not exposed to these drugs. Median time to death was 9.7 years in the exposed group and 18.4 years in the unexposed group, and exposure to these medications significantly increased mortality. Thus, exposure to VGSC-inhibiting drugs associates with reduced survival in breast, bowel and prostate cancer patients. This finding is not consistent with the preclinical data. Despite the strengths of this study including the large sample size, the study is limited by missing information on potentially important confounders such as cancer stage, co-morbidities and cause of death. Further research, which is able to account for these confounding issues, is needed to investigate the relationship between VGSC-inhibiting drugs and cancer survival.

Metastasis is responsible for 90% of cancer deaths^1^. Metastatic tumours are rarely curable and there is an urgent need to identify new molecular targets and develop novel therapies. Voltage-gated sodium channels (VGSCs) regulate electrical excitability and neuronal migration in the central nervous system^2^,^3^,^4^,^5^,^6^,^7^,^8^. Aberrant function of VGSCs is a contributing factor in pathologies including epilepsy, cardiac arrhythmias, and neuropathic pain^9^,^10^. Several commonly prescribed antiepileptic drugs and Class I antiarrhythmic drugs are potent VGSC inhibitors^9^.

VGSCs are also expressed in cells traditionally classed as “non-excitable”, including microglia, astrocytes, immune cells, fibroblasts, and cancer cells, regulating motility and invasion^11^,^12^. The VGSC-inhibiting drugs ranolazine and phenytoin reduce metastasis in preclinical *in vivo* tumour models^13^,^14^. In addition, several VGSC-inhibiting drugs, including carbamazepine, flecainide, mexiletine, phenytoin, riluzole, and valproate, reduce *in vitro* cell behaviours associated with metastasis^15^,^16^,^17^,^18^. Furthermore, valproate, which is also a histone deacetylase inhibitor, is being studied as a potential adjuvant therapy for advanced cancers^19^,^20^,^21^. Together, these data suggest that VGSCs promote invasion and dissemination of metastatic tumour cells, and may represent novel therapeutic targets for slowing/delaying metastasis. Our hypothesis was that use of VGSC-inhibiting drugs before and during a cancer diagnosis may reduce tumour invasion and metastasis, thus improving survival. To test this hypothesis, we analysed data from a UK cohort of cancer patients comparing mortality of those who had a recorded prescription for a VGSC inhibitor prior to their diagnosis with mortality among those who had not. We focused on carcinomas of the breast, bowel and prostate because VGSC expression has been studied extensively in these cancers, and they are the most common^12^,^18^,^22^,^23^,^24^,^25^,^26^,^27^,^28^,^29^.

## Results

**Figure 1:**
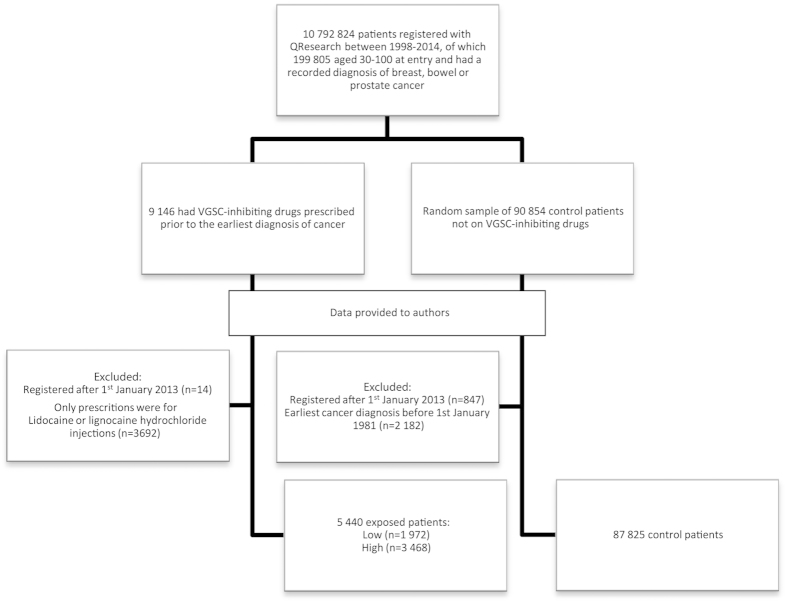
Selection of patients for inclusion in the study. Records provided by QResearch included those of 100 000 patients with breast, bowel, or prostate cancer. Of these, 9,146 had prescription for a VGSC-inhibiting drug prior to cancer diagnosis. Following the indicated exclusions, there were 87,825 unexposed patients and 5,440 exposed patients.

In total 93,265 patients were followed from their date of cancer diagnosis. There were 5,440 patients in the exposed group and 87,825 in the unexposed group (Fig. 1; Table 1). Formal comparisons indicated that there were statistically significant differences between the two groups on all available characteristics (p < 0.001, except age p = 0.05); however, visual inspection suggests these differences are small.

### Overall Survival

**Figure 2:**
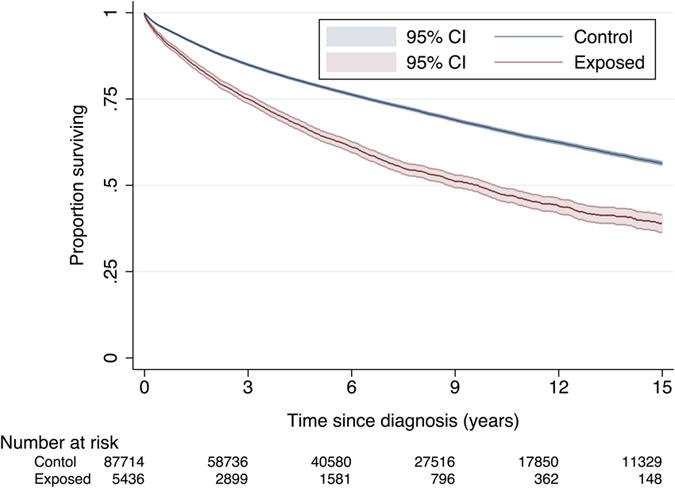
Kaplan-Meier survival curves for unexposed cancer patients (n = 87,714) and those exposed to VGSC-inhibiting drugs (n = 5,436). Log-rank test statistic 613.0, df = 1, p < 0.001. The plot is curtailed at 15 years when around 12% of patients remained in follow-up^54^.

Right censoring occurred at the date the patient left the QResearch practice (n = 14,714) or at date of last data transfer from the GP practice (n = 49,633). The median length of follow-up after diagnosis (to patient’s exit date) was 5.2 years (range 0 days to 33 years). During 658,399 person-years of follow-up, there were 28,918 deaths: 1,995 (37%) in the exposed group and 26,923 (31%) in the unexposed group (Table 2). The median time to death was 9.7 years (95% confidence interval (CI) 8.8–10.2) in the exposed group and 18.4 years (95% CI 18.1–18.7) in the unexposed group (log-rank test statistic 613.0, df = 1, p < 0.001; Fig. 2). The multivariable-adjusted hazard ratio (HR) for death from any cause among VGSC-inhibiting drug users, compared to patients who had never used VGSC-inhibitors, was 1.42 (95% CI 1.36–1.49, p < 0.001), indicating a statistically significant increased risk of death in the exposed group.

The Grambsch and Therneau test indicated potential non-proportionality of hazards for type of cancer, age and age squared (p < 0.05). Log-log and scaled Schoenfeld residual plots are in Supplementary Fig. 1. A sensitivity analysis removing these variables from the model produced a larger HR for the exposed vs. unexposed groups of 1.70 (95% CI 1.62–1.78, p < 0.001). The additional sensitivity analysis including ethnicity, alcohol consumption, smoking status and BMI in the list of original covariates (cancer type, gender, age and age squared) gave a similar HR of 1.48 (95% CI 1.42–1.55, p < 0.001) and so the conclusion did not alter.

All exposed patients had a recorded prescription for a VGSC-inhibiting drug prior to their index cancer diagnosis date and almost half (n = 2,480, 46%) also had a prescription dated after their cancer diagnosis, indicating continuation of their prescription independent of cancer. There was no statistically significant difference in hazard rates between patients who had prescriptions before and after diagnosis compared to those who only had prescriptions before (HR 0.98, 95% CI 0.90–1.07, p = 0.66).

To attempt to capture which patients were actively taking a VGSC-inhibiting drug whilst they had cancer, we identified patients in the exposed group who had a prescription in the year following their diagnosis (n = 2,251, 41%). Findings were similar to the primary result when this group was compared with the unexposed group (HR 1.48, 95% CI 1.39–1.59, p < 0.001).

A Cox regression in which the exposed group was restricted to those patients who started their prescriptions at least one year prior to diagnosis (n = 4,795, 88%) yielded an adjusted HR of 1.43 (95% CI 1.36–1.50, p < 0.001). This analysis aims to exclude patients who may have developed epilepsy as a result of brain metastasis and who started antiepileptic medication as a result of metastatic disease in the 12 months prior to their actual cancer diagnosis.

### Survival Stratified By Cancer Type

Median time to death for patients whose first diagnosis of one of the three index cancers was: prostate cancer, 11.2 years (95% CI 10.9–11.5); bowel cancer, 11.9 years (95% CI 11.4–12.3); and breast cancer, 24.3 years (95% CI 23.8–24.7). Separate Cox proportional hazards models comparing the exposed and unexposed groups within these subgroups gave a HR of 1.64 (95% CI 1.51–1.77, p < 0.001) among breast cancer patients; 1.41 (95% CI 1.31–1.52, p < 0.001) among bowel cancer patients; and 1.23 (95% CI 1.13–1.33, p < 0.001) among prostate cancer patients (males only so gender was omitted as a covariate) (Table 2).

### Survival By Level Of Drug Exposure And Drug Type

In total, 1,972 (36%) exposed patients were classified in the low exposure group and 3,468 (64%) in the high exposure group. The HR was 1.28 (95% CI 1.19–1.39, p < 0.001) when the low exposure group was compared with the unexposed group and 1.50 (95% CI 1.42–1.59, p < 0.001) when the high exposure group was compared with the unexposed group (Table 2). The high exposure group had a HR 1.17 times higher than the low exposure group (95% CI 1.07–1.28, p = 0.001).

Patients with cancer who were using VGSC-inhibiting medication regularly had a higher risk of death than unexposed patients with cancer (HR 1.64, 95% CI 1.54–1.76, p < 0.001), as did non-regular users with cancer (HR 1.29, 95% CI 1.21–1.37, p < 0.001).

We stratified patients based on the most common drug they had been prescribed and compared each group with the unexposed group in turn: other Class I antiarrhythmic drug (n = 849, HR 1.11, 95% CI 0.98–1.24, p = 0.09); lamotrigine (n = 166, HR 1.32, 95% CI 0.96–1.82, p = 0.08); carbamazepine (n = 2,727, HR 1.38, 95% CI 1.29–1.47, p < 0.001); phenytoin (n = 719, HR 1.67, 95% CI 1.50–1.86, p < 0.001); and valproate (n = 903, HR 1.74, 95% CI 1.56–1.93, p < 0.001) (Table 2). The other drug classes consisted of too few individuals to be included in a comparison (topiramate, n = 64; ranolazine, n = 9; riluzole, n = 2; lacosamide, n = 1).

### Survival From Any Cancer

Of those included in the population for analysis, 3,773 had a diagnosis for a different cancer prior to their diagnosis of one of the three index cancers: 312 (6%) in the exposed group and 3,461 (4%) in the unexposed group. Mortality from date of first cancer diagnosis was significantly increased in the exposed group relative to the unexposed group (HR 1.39, 95% CI 1.33–1.46, p < 0.001).

## Discussion

Our results indicate that the survival of breast, bowel, and prostate cancer patients prescribed VGSC-inhibiting drugs is significantly reduced compared to cancer patients not taking these drugs. These drugs have well-documented safety profiles, reviewed in detail elsewhere^30^,^31^. Adjustment for ethnicity, BMI, alcohol consumption, and smoking status did not alter this conclusion. The impact of drug intake on survival is worse for breast and bowel cancer patients than for prostate cancer patients. It is not possible to establish whether this is a cancer-specific effect or is confounded by gender (females account for 98% of the breast cancer patients, 46% of bowel cancer patients and 0% of prostate cancer patients). The HR for patients with high exposure to the index drugs is larger than for those with low exposure. Survival is significantly reduced for patients prescribed carbamazepine, valproate and phenytoin, but not for those prescribed lamotrigine or other Class I antiarrhythmics.

Our findings contrast with preclinical studies showing that VGSC inhibition reduces invasion, migration and metastasis^13^,^14^,^15^,^16^,^17^,^18^,^20^,^32^. A limitation of these preclinical studies is that they have focused on tumour cells in isolation without consideration for the potential detrimental effect of VGSC-inhibiting drugs on the immune system or drug-drug interaction with chemotherapy^33^,^34^. Our results also contrast with several recent clinical studies that suggest that valproate may improve outcome in patients with several types of cancer^19^,^35^,^36^. The results of these studies are generally accepted to be due to the function of valproate as a histone deacetylase inhibitor, rather than as a VGSC inhibitor^20^,^21^,^37^. One major difference between our study and the design of these trials is that we included only patients who had started taking valproate prior to cancer diagnosis, whereas clinical trials typically added valproate as second line therapy in patients with advanced disease. We were unable to determine which patients were actively taking VGSC-inhibiting medication whilst they had cancer. Therefore, as a best estimate, we identified patients who had a recorded prescription in the year following cancer diagnosis, and found that these patients still had a significantly increased mortality. However, we did not have access to indication for VGSC-inhibiting drug prescriptions or cause of death and further work is required to establish why cancer patients exposed to VGSC-inhibiting drugs have a shorter survival time.

Studies in animals have suggested that several VGSC-inhibiting antiepileptic drugs, including phenytoin, valproate and carbamazepine, may increase risk of developing certain cancers^38^, although epidemiological data are conflicting^39^,^40^,^41^. In addition, no relationship between these drugs and survival of cancer patients has previously been reported. Several antiepileptic drugs, including phenytoin and carbamazepine, are potent inducers of hepatic drug-metabolising enzymes^33^. Indeed, a number of cancer chemotherapeutic agents are substrates for enzyme-inducing action of antiepileptic drugs, including phenytoin and carbamazepine^38^. It is therefore possible that the reduced survival of breast, bowel, and prostate cancer patients exposed to VGSC-inhibiting drugs identified in our study may be due to reduced bioavailability and efficacy of concurrent chemotherapies as a result of hepatic enzyme induction. However, we also found reduced survival of cancer patients taking valproate, which has no reported enzyme-inducing activity^33^. Our data suggest that the suitability of valproate in cancer patients should be studied further.

A weakness of this study is that the estimation of true association may be affected by confounding by indication. Epilepsy patients have an increased risk of premature mortality, predominant at younger age and shortly after diagnosis, but can also persist for decades after index seizure. For example, all cause standardised mortality ratio of 2.2–3.2 and HR of 3.2 have been reported in several cohort studies^42^,^43^,^44^. Although seizures can contribute to mortality, several comorbidities may interact with epilepsy, worsening outcome, including psychiatric diseases, gynaecological disorders, accidents, and cancer^45^. It is possible that the reduced survival of breast, bowel, and prostate cancer patients exposed to VGSC-inhibiting drugs is due to the majority of these patients having epilepsy as their initial diagnosis and a poorer general health status^46^. In addition to epilepsy, the index drugs may also have been prescribed for other, unrelated conditions, including bipolar disorder, depression, migraine and chronic pain^9^. Thus, it is not clear whether co-morbidities of patients exposed to these drugs contributed to their reduced survival. Finally, it is possible that some patients had epilepsy caused by brain metastases prior to index cancer diagnosis. In such cases, prescription of VGSC-inhibiting antiepileptic drugs might seem to be associated with reduced survival, although metastasis itself had been the cause. However, survival was still significantly decreased in the intervention arm when we tried to control for this by only including patients who had been on VGSC-inhibiting drugs starting ≥12 months before first cancer diagnosis.

The strength of this prospectively designed cohort study is that it uses primary care data and thus contains a large sample size and statistical power. However, there are a number of weaknesses to take into consideration, including the use of GP diagnosis codes to identify cancer and death, the use of prescription records to identify medication use when non-compliance is possible, and the lack of information on cause of death. A further limitation is that the number of cases exposed to VGSC-inhibiting drugs was relatively small. In addition, we did not have information on several potential confounders, including epilepsy prevalence, reason for VGSC-inhibiting drug prescription, and tumour stage. Finally, it was necessary to use overall survival as a surrogate indicator of metastasis because metastasis and cause of death are not reliably recorded in general practice data.

In conclusion, we set out to test the hypothesis that breast, bowel and prostate cancer patients who had prolonged prescription of VGSC-inhibiting drugs lived longer than those not taking this medication. To our surprise our findings indicate the opposite, contrasting with the preclinical data^13^,^14^,^15^,^16^,^17^,^18^,^32^. The reasons for this contradiction are not yet clear. Further work is needed to define cancer-specific mortality risk by drug type, dosing and duration, controlling for co-morbidity and indication.

## Methods

### Ethical Approval

This study was approved by the Department of Biology Ethical Review Body at the University of York. The methods were carried out in accordance with the approved guidelines. The protocol has been published^47^.

### Data Source

The data source was QResearch, a large consolidated UK primary care database derived from the pseudonymised health records of >13 million patients from 754 general practices. QResearch holds data from patients who are currently registered with practices as well as patients who may have died or left. The database has been validated with other sources of information^48^,^49^,^50^.

### Study Population

An open cohort of patients registered with a QResearch general practice during the study period (01/01/98–31/12/13), aged 30 years or older when they joined, and who had a diagnosis of breast, bowel, or prostate cancer (referred to as the *index* cancers) (n = 10,792,824). From this cohort, patients who had a recorded prescription for a VGSC-inhibiting drug prior to the earliest diagnosis of an index cancer were identified (n = 9,146). A randomly generated sample of 90,854 patients with breast, bowel, or prostate cancer who did not have a prescription for a VGSC-inhibiting drug made up the unexposed group. A final dataset of 100,000 patients was provided to the authors (Fig. 1).

Data were provided on year of birth, sex, ethnicity, date of entry to the QResearch database, age at entry, exit date (earliest of leaving the QResearch practice, death or latest data transfer from GP), status at exit date (died, still registered or left), cancer diagnosis, date of diagnosis, date of death, BMI, alcohol consumption, smoking status, and prescriptions of the index VGSC-inhibiting drugs (carbamazepine, lacosamide, lamotrigine, phenytoin, ranolazine, riluzole, topiramate, valproic acid/sodium valproate, and other Class I antiarrhythmics (disopyramide, flecainide, lidocaine, mexiletine, procainamide, propafenone, quinidine)). Cancer diagnoses, ethnicity, alcohol intake, and use of cigarettes/tobacco were classified according to Read codes (Supplementary Table 1)^51^. We had no access to any data on other prescriptions, indication for prescriptions, co-morbidities, cancer stage, other treatments or therapies received (including cancer treatments such as chemotherapy or radiotherapy), cancer recurrence, or cause of death.

We excluded patients registered with QResearch after 31^st^ December 2012 to allow at least one year’s follow-up for all patients (n = 861). The earliest dates of cancer diagnosis were much later in the exposed group (from 1981) than the unexposed group (from 1940); therefore, we excluded any patient whose primary index cancer diagnosis was before 01/01/81 (n = 2,182). Anomalous, incorrect or infeasible dates were removed. Dates of cancer diagnosis indicating the patient was <25 at time of diagnosis were excluded.

### Potential Confounders

Ethnicity was categorised according to the 2011 UK census. Missing data techniques were not implemented; instead, a ‘not recorded/known’ category was used. For potential confounders (alcohol, smoking, BMI), the last value recorded prior to cancer diagnosis was used. If a value was only recorded after cancer diagnosis then the earliest was taken. Using Read codes, or raw values where the Read code was uninformative, risk factors were categorised as follows: alcohol consumption - non/trivial drinker (<1 unit/day), light drinker (1–2 units/day), moderate-very heavy drinker (3+ units/day), not recorded/known; smoking status - ex-smoker, non/trivial smoker (<1 cigarette (or equivalent) per day), smoker (1+ cigarette/day), not recorded/known; and BMI – underweight (<18.5), normal range (18.5–24), overweight (25–29), obese (30+), not recorded/known.

### VGSC-Inhibiting Drug Use

All prescriptions relating to one of the VGSC-inhibiting drugs were provided. One-off prescriptions for lidocaine injections were excluded as these were likely used as a local anaesthetic and therefore to have a transient effect (n = 3,692). Extent of drug exposure was estimated by calculating the time between the first and last recorded prescription. Patients were classified into two exposure groups: low (<6 months, n = 1,972), and high (≥6 six months, n = 3,468). In addition, the most commonly prescribed class of drug for each patient was identified.

Patients who had at ≥2 prescriptions relating to one of the VGSC-inhibiting drugs within 2 years before the date of the cancer diagnosis, including at least one within 6 months before were classified as regular and recent VGSC-inhibiting drug users (n = 2,153). All other patients exposed to the index drugs were classified as non-regular users (n = 3,287)^52^.

### Statistical Analysis

Characteristics of the exposed and unexposed groups are summarised using descriptive statistics (mean, SD, median, minimum, maximum) for continuous variables, and count and percentage for categorical data. Formal statistical comparisons were made between the two groups using a t-test or chi-squared test as appropriate.

Analyses were conducted in Stata v13 using two-sided statistical tests at the 5% significance level. Regression models to compare the exposed and unexposed groups were adjusted for type of cancer, gender and age at diagnosis (age was included as both a linear and quadratic term (age+age^2^)) unless otherwise stated. Multivariable-adjusted HRs are presented with 95% CIs and p-values. The distribution of time from cancer diagnosis to death (all cause) was described using Kaplan-Meier survival estimates for the two groups. The statistical equivalence of the two curves was examined using the log-rank test. Survival from cancer diagnosis was compared between the exposed and unexposed groups using Cox proportional hazards regression. In a sensitivity analysis, we also included ethnicity, smoking status, alcohol consumption and BMI as covariates.

Secondary analyses using separate multivariable-adjusted Cox models compared the survival of the unexposed group with cancer with the following subsets of patients from the exposed group with cancer: the low exposure group; the high exposure group; regular VGSC users; non-regular VGSC users; patients who had at least one prescription in the year following their diagnosis; and, for each drug in turn, patients for whom that drug was the most commonly prescribed. We also considered survival stratified by cancer type using the same model specification but omitting cancer type as a covariate (and gender among prostate cancer patients). For most patients, one of the index cancers was the primary cancer diagnosis, but a small number were diagnosed with a different cancer first. We therefore compared survival from the date of first cancer diagnosis with a Cox model, adjusting for type of cancer (including ‘Other’ as a category in the cancer type variable), gender, age and age^2^.

Cox regression models require a number of assumptions to be valid. Firstly, the issue of non-informative censoring. Individuals in this study were censored if they left the QResearch practice or their date of last data transfer from the GP practice was before death. These mechanisms for censoring are not related to the probability of death so the assumption is reasonable. The second key assumption is that the proportional hazards model applies. We used the Grambsch and Therneau test (*estat phtest* command with the *detail* option in Stata) to assess the proportionality for each predictor and for the model as a whole^53^. In addition, we produced log-log plots (–log(-log(S(t))) against log(time), where S(t) is the survivor function at time *t*) for the categorical predictors, and scaled Schoenfeld residuals against time for the continuous factors (age and age squared).

## Additional Information

**How to cite this article**: Fairhurst, C. *et al.* Sodium channel-inhibiting drugs and survival of breast, colon and prostate cancer: a population-based study. *Sci. Rep.* **5**, 16758; doi: 10.1038/srep16758 (2015).

## Supplementary Material

Supplementary Information

## Figures and Tables

**Table 1:** Patient characteristics by VGSC-inhibiting exposure group.

Characteristic^1^	Unexposed^2^ (n = 87825)	Exposed^3^ (n = 5440)	Total (n = 93265)
Gender, n (%)			
* Male*	37684 (42. 9)	2585 (47.5)	40269 (43.2)
* Female*	50141 (57.1)	2855 (52.5)	52996 (56.8)
Age			
* Mean* (*SD*)	63.5 (13.7) 64	63.9 (12.7) 65	63.5 (13.6)
* Median* (*min, max*)	(30, 99)	(30, 98)	64 (30, 99)
Ethnicity, n (%)			
* White*	50588 (57.6)	3330 (61.2)	53918 (57.8)
* Mixed/Multiple ethnic groups*	321 (0.4)	15 (0.3)	336 (0.4)
* Asian/Asian British*	1095 (1.2)	34 (0.6)	1129 (1.2)
* Black/Black British*	1374 (1.6)	43 (0.8)	1417 (1.5)
* Other*	208 (0.2)	4 (0.1)	212 (0.2)
* Not recorded/known*	34239 (39.0)	2014 (37.0)	36253 (38.9)
Smoking status, n (%)			
* Smoker*	12547 (14.3)	804 (14.8)	13351 (14.3)
* Non/trivial smoker*	46614 (53.1)	2767 (50.9)	49381 (52.9)
* Ex-smoker*	23409 (26.7)	1667 (30.6)	25076 (26.9)
* Not recorded/known*	5255 (6.0)	202 (3.7)	5457 (5.9)
* *Alcohol consumption, n (%)			
* Moderate-heavy drinker*	6345 (7.2)	363 (6.7)	6708 (7.2)
* Light drinker*	18772 (21.4)	1019 (18.7)	19791 (21.2)
* Non/trivial drinker*	51037 (58.1)	3519 (64.7)	54556 (58.5)
* Not recorded/known*	11671 (13.3)	539 (9.9)	12210 (13.1)
BMI category, n (%)			
* Obese*	15631 (17.8)	1182 (21.7)	16813 (18.0)
* Overweight*	30495 (34.7)	1932 (35.5)	32427 (34.8)
* Normal range*	30217 (34.4)	1740 (32.0)	31957 (34.3)
* Underweight*	541 (0.6)	40 (0.7)	581 (0.6)
* Not recorded/known*	10941 (12.5)	546 (10.0)	11487 (12.3)
Type of cancer^4^, n (%)			
* Breast*	41342 (47.1)	2230 (41.0)	43572 (46.7)
* Bowel*	21349 (24.3)	1477 (27.2)	22826 (24.5)
* Prostate*	25134 (28.6)	1733 (31.9)	26867 (28.8)
Exit status, n (%)			
* Died*	26923 (30.7)	1995 (36.7)	28918 (31.0)
* Left*	14044 (16.0)	670 (12.3)	14714 (15.8)
* Still registered*	46858 (53.4)	2775 (51.0)	49633 (53.2)

^1^Differences between groups p < 0.001 (except age p = 0.05).

^2^Breast/bowel/prostate cancer patients not exposed to VGSC-inhibiting drugs.

^3^Cancer patients exposed to VGSC-inhibiting drugs for any duration.

^4^First instance of one of reference cancers.

**Table 2:** Time to death estimates by group.

	Unexposed^1^ (n = 87825)	Exposed^2^ (n = 5440)
**Overall survival**		
Number of events (deaths), n (%)	26923 (30.7)	1995 (36.7)
Median time to death, years (95% CI)	18.4 (18.1 to 18.7)	9.7 (8.8 to 10.2)
Log-rank test statistic, p-value	613.0, df = 1, p < 0.001	
Adjusted analysis, HR (95% CI, p-value)^3^	1.42 (1.36 to 1.49, p < 0.001)	
**Survival by type of cancer**^4^	**HR (95% CI, p-value)**	
*Prostate* [Unexposed (n = 25134) vs exposed (n = 1733)]	1.23 (1.13 to 1.33, p < 0.001)	
*Bowel* [Unexposed (n 21349) vs exposed (n = 1477)]	1.41 (1.31 to 1.52, p < 0.001)	
*Breast* [Unexposed (n = 41342) vs exposed (n = 2230)]	1.64 (1.51 to 1.77, p < 0.001)	
**Survival by drug exposure**^5^	**HR (95% CI, p-value)**	
Unexposed (n = 87825) vs low exposed (n = 1972)	1.28 (1.18 to 1.38, p < 0.001)	
Unexposed (n = 87825) vs high exposed (n = 3468)	1.50 (1.42 to 1.59, p < 0.001)	
**Survival by most common drug prescription**	**HR (95% CI, p-value)**	
Unexposed (n = 87825) vs exposed [Class I antiarrhythmic] (n = 849)	1.11 (0.98 to 1.24, p = 0.09)	
Unexposed (n = 87825) vs exposed [Lamotrigine] (n = 166)	1.32 (0.96 to 1.82, p = 0.08)	
Unexposed (n = 87825) vs exposed [Carbamazepine] (n = 2727)	1.38 (1.29 to 1.47, p < 0.001)	
Unexposed (n = 87825) vs exposed [Phenytoin] (n = 719)	1.67 (1.50 to 1.86, p < 0.001)	
Unexposed (n = 87825) vs exposed [Valproate] (n = 903)	1.74 (1.56 to 1.93, p < 0.001)	

^1^Breast/bowel/prostate cancer patients not exposed to VGSC-inhibiting drugs.

^2^Cancer patients exposed to VGSC-inhibiting drugs for any duration.

^3^All hazard ratios (HR; with 95% confidence interval and p-value) compare unexposed and exposed groups. A HR >1 indicates exposed group has increased mortality relative to unexposed group. Models are adjusted for cancer type, gender, age and age as a quadratic term except model.

^4^for which cancer type is omitted (gender also omitted in the model among prostate cancer patients).

^5^Low exposure, <6 months; high exposure, ≥6 months.
