# Supplementary material for: Sodium channel-inhibiting drugs and survival of breast, colon and prostate cancer: a population-based study
Source: Sci Rep. 2015 Nov 18;5:16758. doi: 10.1038/srep16758 (PMC4649474; doi:10.1038/srep16758)
Supplement: Supplementary Information [file srep16758-s1.pdf]

Supplementary Data

Sodium channel-inhibiting drugs and survival of breast, colon and prostate cancer: a population-based study

Caroline Fairhurst, MSc[a], Ian Watt, MBChB[a,b], Fabiola Martin, MBChB[b,c], Martin Bland, PhD[a], and William J. Brackenbury, PhD[c*]

[a]Department of Health Sciences, University of York, York, UK, YO10 5DD

[b]Hull York Medical School, York, UK, YO10 5DD

[c]Department of Biology, University of York, York, UK, YO10 5DD

*Corresponding author:

William J. Brackenbury, PhD

Department of Biology, University of York, Heslington, York, YO10 5DD, UK

E-mail: william.brackenbury@york.ac.uk

Telephone: +44 (0) 1904 328 284

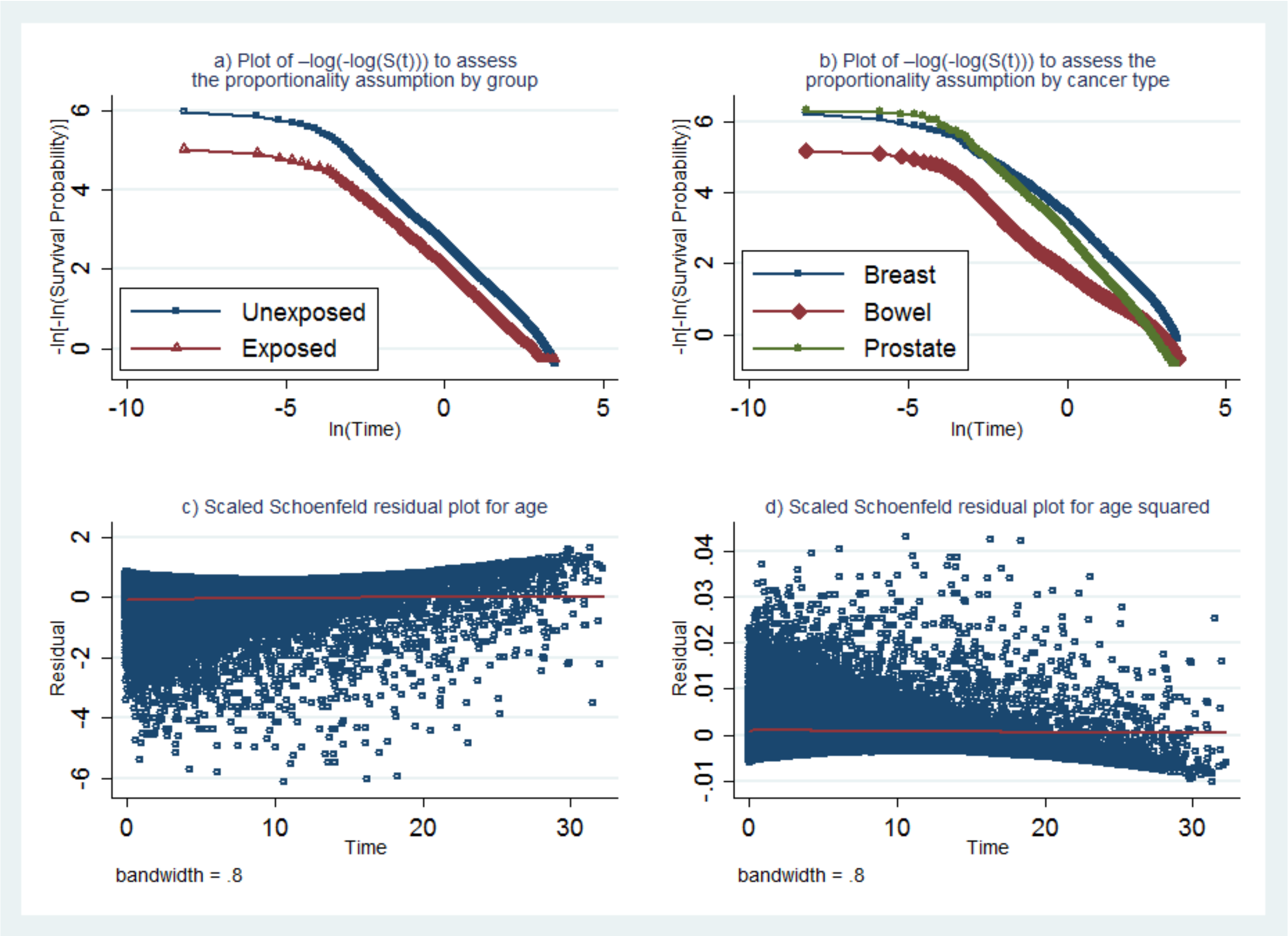

Supplementary Fig. 1. Log-log and scaled Schoenfeld residual plots. (a) and (b): log-log plots for group (exposed vs unexposed) and cancer type, repsectively. Non-parallel lines indicate non-proportionality of the hazards. A log-log plot is not displayed for gender since there was no evidence of non-proportionality for this variable determined by the Grambsch and Therneau test. (c) and (d): Schoenfeld residuals versus time for the age and age-squared covariates, respectively. Deviation of the red line from a horizontal line at y=0 indicates that there is violation of the proportionality assumption.

Supplementary Table 1. List of Read Codes used in this study

Group Name	Read code	Description
Breast cancer	B34	Malignant neoplasm of female breast
Breast cancer	B34-1	Ca female breast
Breast cancer	B34-98	Ca breast - NOS
Breast cancer	B34-99	Carcinoma breast
Breast cancer	B340	Malignant neoplasm of nipple and areola of female breast
Breast cancer	B340-99	Ca breast - nipple/central
Breast cancer	B3400	Malignant neoplasm of nipple of female breast
Breast cancer	B3401	Malignant neoplasm of areola of female breast
Breast cancer	B340z	Malignant neoplasm of nipple or areola of female breast NOS
Breast cancer	B341	Malignant neoplasm of central part of female breast
Breast cancer	B342	Malignant neoplasm of upper-inner quadrant of female breast
Breast cancer	B342-99	Ca breast-upper,inner quadrant
Breast cancer	B343	Malignant neoplasm of lower-inner quadrant of female breast
Breast cancer	B343-99	Ca breast-lower,inner quadrant
Breast cancer	B344	Malignant neoplasm of upper-outer quadrant of female breast
Breast cancer	B344-99	Ca breast-upper,outer quadrant
Breast cancer	B345	Malignant neoplasm of lower-outer quadrant of female breast
Breast cancer	B345-99	Ca breast-lower,outer quadrant
Breast cancer	B346	Malignant neoplasm of axillary tail of female breast
Breast cancer	B346-99	Ca breast - axillary tail
Breast cancer	B347	Malignant neoplasm, overlapping lesion of breast
Breast cancer	B34y	Malignant neoplasm of other site of female breast
Breast cancer	B34y0	Malignant neoplasm of ectopic site of female breast
Breast cancer	B34yz	Malignant neoplasm of other site of female breast NOS
Breast cancer	B34z	Malignant neoplasm of female breast NOS
Breast cancer	B34z-99	Ca breast - NOS
Breast cancer	B35	Malignant neoplasm of male breast
Breast cancer	B35-99	Ca breast - male
Breast cancer	B350	Malignant neoplasm of nipple and areola of male breast
Breast cancer	B3500	Malignant neoplasm of nipple of male breast
Breast cancer	B3501	Malignant neoplasm of areola of male breast
Breast cancer	B350z	Malignant neoplasm of nipple or areola of male breast NOS
Breast cancer	B35z	Malignant neoplasm of other site of male breast
Breast cancer	B35z0	Malignant neoplasm of ectopic site of male breast
Breast cancer	B35zz	Malignant neoplasm of male breast NOS
Breast cancer	B36	Local recurrence of malignant tumour of breast
Breast cancer	B58y0	Secondary malignant neoplasm of breast
Breast cancer	B830	Carcinoma in situ of breast
Breast cancer	B830-99	Carcinoma in situ breast
Breast cancer	B8300	Lobular carcinoma in situ of breast
Breast cancer	B8301	Intraductal carcinoma in situ of breast
Breast cancer	B933	Neoplasm of uncertain behaviour of breast
Breast cancer	BA03	Neoplasm of unspecified nature of breast
Breast cancer	BB94-1	[M]Secretory breast carcinoma
Breast cancer	Byu6	[X]Malignant neoplasm of breast
Breast cancer	EMISNQLO13	Local recurrence of malignant tumour of breast
Breast cancer	HNG0190	[RFC] Breast cancer
Breast cancer	ZV103	[V]Personal history of malignant neoplasm of breast

Group Name	Read code	Description
Colorectal cancer	B13	Malignant neoplasm of colon
Colorectal cancer	B130	Malignant neoplasm of hepatic flexure of colon
Colorectal cancer	B130-99	Ca hepatic flexure - colon
Colorectal cancer	B131	Malignant neoplasm of transverse colon
Colorectal cancer	B131-99	Ca transverse colon
Colorectal cancer	B132	Malignant neoplasm of descending colon
Colorectal cancer	B132-99	Ca descending colon
Colorectal cancer	B133	Malignant neoplasm of sigmoid colon

Colorectal cancer	B133-99	Ca sigmoid colon
Colorectal cancer	B134	Malignant neoplasm of caecum
Colorectal cancer	B134-1	Carcinoma of caecum
Colorectal cancer	B135	Malignant neoplasm of appendix
Colorectal cancer	B136	Malignant neoplasm of ascending colon
Colorectal cancer	B136-99	Ca ascending colon
Colorectal cancer	B137	Malignant neoplasm of splenic flexure of colon
Colorectal cancer	B137-99	Ca splenic flexure - colon
Colorectal cancer	B138	Malignant neoplasm, overlapping lesion of colon
Colorectal cancer	B139	Hereditary nonpolyposis colon cancer
Colorectal cancer	B13y	Malignant neoplasm of other specified sites of colon
Colorectal cancer	B13z	Malignant neoplasm of colon NOS
Colorectal cancer	B13z-1	Colonic cancer
Colorectal cancer	B14	Malignant neoplasm of rectum, rectosigmoid junction and anus
Colorectal cancer	B14-99	Ca rectum + Ca anus
Colorectal cancer	B140	Malignant neoplasm of rectosigmoid junction
Colorectal cancer	B140-99	Ca rectosigmoid junction
Colorectal cancer	B141	Malignant neoplasm of rectum
Colorectal cancer	B141-1	Carcinoma of rectum
Colorectal cancer	B141-2	Rectal carcinoma
Colorectal cancer	B141-99	Adenocarcinoma of rectum
Colorectal cancer	B1420	Malignant neoplasm of cloacogenic zone
Colorectal cancer	B143	Malignant neoplasm of anus unspecified
Colorectal cancer	B14y	Malig neop other site rectum, rectosigmoid junction and anus
Colorectal cancer	B14z	Malignant neoplasm rectum,rectosigmoid junction and anus NOS
Colorectal cancer	B14z-99	Ca rectum + Ca anus NOS
Colorectal cancer	B1z0-1	Cancer of bowel
Colorectal cancer	B575	Secondary malignant neoplasm of large intestine and rectum
Colorectal cancer	B5750	Secondary malignant neoplasm of colon
Colorectal cancer	B5751	Secondary malignant neoplasm of rectum
Colorectal cancer	B575z	Secondary malig neop of large intestine or rectum NOS
Colorectal cancer	B803	Carcinoma in situ of colon
Colorectal cancer	B8030	Carcinoma in situ of hepatic flexure of colon
Colorectal cancer	B8031	Carcinoma in situ of transverse colon
Colorectal cancer	B8032	Carcinoma in situ of descending colon
Colorectal cancer	B8033	Carcinoma in situ of sigmoid colon
Colorectal cancer	B8036	Carcinoma in situ of ascending colon
Colorectal cancer	B8037	Carcinoma in situ of splenic flexure of colon
Colorectal cancer	B8038	High grade dysplasia of colon
Colorectal cancer	B803z	Carcinoma in situ of colon NOS
Colorectal cancer	B804	Carcinoma in situ of rectum and rectosigmoid junction
Colorectal cancer	B8040	Carcinoma in situ of rectosigmoid junction
Colorectal cancer	B8041	Carcinoma in situ of rectum
Colorectal cancer	B804z	Carcinoma in situ of rectum or rectosigmoid junction NOS
Colorectal cancer	B902	Neop of uncertain behaviour stomach, intestines and rectum
Colorectal cancer	B9024	Neoplasm of uncertain behaviour of colon
Colorectal cancer	B9025	Neoplasm of uncertain behaviour of rectum
Colorectal cancer	B902z	Neop of uncertain behaviour stomach, intestine or rectum NOS
Colorectal cancer	BB5N	[M]Adenomatous and adenocarcinomatous polyps of colon
Colorectal cancer	EGTON2B152	Cause of Death- Ca Rectum
Colorectal cancer	EMISNQHE41	Hereditary nonpolyposis colon cancer
Colorectal cancer	HNG0201	[RFC] Cancer of the rectum

**Group Name**	**Read code**	**Description**
Prostate cancer	14270	H/O: prostate cancer
Prostate cancer	1J08	Suspected prostate cancer
Prostate cancer	4M0	Gleason grading of prostate cancer
Prostate cancer	4M00	Gleason prostate grade 2-4 (low)
Prostate cancer	4M01	Gleason prostate grade 5-7 (medium)
Prostate cancer	4M02	Gleason prostate grade 8-10 (high)
Prostate cancer	B46	Malignant neoplasm of prostate

Prostate cancer	B58y5	Secondary malignant neoplasm of prostate
Prostate cancer	B7C20	Adenoma of prostate
Prostate cancer	B834	Carcinoma in situ of prostate
Prostate cancer	B8340	High grade prostatic intraepithelial neoplasia
Prostate cancer	B8341	Prostatic intraepithelial neoplasia
Prostate cancer	B915	Neoplasm of uncertain behaviour of prostate
Prostate cancer	EMISNQPR140	Prostatic intraepithelial neoplasia
Prostate cancer	HNG0200	[RFC] Cancer of the prostate
Prostate cancer	K223	Dysplasia of prostate
Prostate cancer	PCSDT1PR16	Prostate carcinoma
Prostate cancer	ZV104-5	[V]Personal history of malignant neoplasm of prostate

Group Name	Read code	Description
Alcohol status	136	Alcohol consumption
Alcohol status	1361	Teetotaller
Alcohol status	1361-1	Non drinker alcohol
Alcohol status	1361-2	Non-drinker alcohol
Alcohol status	1362	Trivial drinker - <1u/day
Alcohol status	1362-1	Drinks rarely
Alcohol status	1362-2	Drinks occasionally
Alcohol status	1363	Light drinker - 1-2u/day
Alcohol status	1364	Moderate drinker - 3-6u/day
Alcohol status	1365	Heavy drinker - 7-9u/day
Alcohol status	1366	Very heavy drinker - >9u/day
Alcohol status	1367	Stopped drinking alcohol
Alcohol status	1368	Alcohol consumption unknown
Alcohol status	1369	Suspect alcohol abuse - denied
Alcohol status	136A	Ex-trivial drinker (<1u/day)
Alcohol status	136a	Increasing risk drinking
Alcohol status	136B	Ex-light drinker - (1-2u/day)
Alcohol status	136b	Feels should cut down drinking
Alcohol status	136C	Ex-moderate drinker - (3-6u/d)
Alcohol status	136c	Higher risk drinking
Alcohol status	136D	Ex-heavy drinker - (7-9u/day)
Alcohol status	136d	Lower risk drinking
Alcohol status	136E	Ex-very heavy drinker-(>9u/d)
Alcohol status	136e	Declines to state current alcohol consumption
Alcohol status	136F	Spirit drinker
Alcohol status	136G	Beer drinker
Alcohol status	136H	Drinks beer and spirits
Alcohol status	136I	Drinks wine
Alcohol status	136J	Social drinker
Alcohol status	136K	Alcohol intake above recommended sensible limits
Alcohol status	136L	Alcohol intake within recommended sensible limits
Alcohol status	136M	Current non drinker
Alcohol status	136N	Light drinker
Alcohol status	136O	Moderate drinker
Alcohol status	136P	Heavy drinker
Alcohol status	136Q	Very heavy drinker
Alcohol status	136R	Binge drinker
Alcohol status	136S	Hazardous alcohol use
Alcohol status	136T	Harmful alcohol use
Alcohol status	136V	Alcohol units per week
Alcohol status	136X	Alcohol units consumed on heaviest drinking day
Alcohol status	136Y	Drinks in morning to get rid of hangover
Alcohol status	136Z	Alcohol consumption NOS
Alcohol status	E23-2	Alcohol problem drinking
Alcohol status	EGTON418	Alcohol intake
Alcohol status	EGTONGR6	Grade A none drinker or rare
Alcohol status	EGTONGR7	Grade B 0-20 units/week
Alcohol status	EGTONGR8	Grade C 21-49 units/week

Alcohol status	EGTONGR9	Grade D 50 or more units/week
Alcohol status	EMISNQUN23	Unsuccessful attempts to reduce alcohol consumption
Alcohol status	EMISQAL1	Alcohol AUDIT score
Alcohol status	EMISQAL2	Alcohol reduction-maintain abstinence
Alcohol status	EMISQAU1	AUDIT score <4 no drinking problem
Alcohol status	EMISQAU2	AUDIT score >5 alcohol dependence syndrome
Alcohol status	EMISQAU3	AUDIT score >5 alcohol problem drinker
Alcohol status	EMISQNO6	Not interested in reducing alcohol
Alcohol status	EMISQOT3	Other alcohol related information
Alcohol status	EMISQRE13	Ready to start reducing alcohol
Alcohol status	EMISQTH4	Thinking about reducing alcohol

Group Name	Read code	Description
BMI	22K	Body Mass Index
BMI	22K1	Body Mass Index normal K/M2
BMI	22K2	Body Mass Index high K/M2
BMI	22K3	Body Mass Index low K/M2
BMI	22K4	Body mass index index 25-29 - overweight
BMI	22K5	Body mass index 30+ - obesity
BMI	22K6	Body mass index less than 20
BMI	22K7	Body mass index 40+ - severely obese
BMI	22K8	Body mass index 20-24 - normal
BMI	22K9	Body mass index centile
BMI	22K90	Baseline body mass index centile
BMI	22KA	Target body mass index
BMI	22KB	Baseline body mass index
BMI	EMISNQBM1	BMI centile
BMI	EMISNQBO29	Body mass index less than 18.5
BMI	EMISNQTA9	Target body mass index
BMI	JHCBO5	Body mass index 18.5-24.9
BMI	PCNQBO1	Body mass index

Group Name	Read code	Description
Ethnicity	226	O/E - ethnic group
Ethnicity	226-1	O/E - ethnic origin
Ethnicity	226Z	O/E - ethnic group NOS
Ethnicity	916E	Patient ethnicity unknown
Ethnicity	918t	Carer from Black and minority ethnic group
Ethnicity	9i	Ethnic category - 2001 census
Ethnicity	9i0	British or mixed British - ethnic category 2001 census
Ethnicity	9i00	White British - ethnic category 2001 census
Ethnicity	9i1	Irish - ethnic category 2001 census
Ethnicity	9i10	White Irish - ethnic category 2001 census
Ethnicity	9i2	Other White background - ethnic category 2001 census
Ethnicity	9i20	English - ethnic category 2001 census
Ethnicity	9i21	Scottish - ethnic category 2001 census
Ethnicity	9i22	Welsh - ethnic category 2001 census
Ethnicity	9i23	Cornish - ethnic category 2001 census
Ethnicity	9i24	Northern Irish - ethnic category 2001 census
Ethnicity	9i25	Ulster Scots - ethnic category 2001 census
Ethnicity	9i26	Cypriot (part not stated) - ethnic category 2001 census
Ethnicity	9i27	Greek - ethnic category 2001 census
Ethnicity	9i28	Greek Cypriot - ethnic category 2001 census
Ethnicity	9i29	Turkish - ethnic category 2001 census
Ethnicity	9i2A	Turkish Cypriot - ethnic category 2001 census
Ethnicity	9i2B	Italian - ethnic category 2001 census
Ethnicity	9i2C	Irish Traveller - ethnic category 2001 census
Ethnicity	9i2D	Traveller - ethnic category 2001 census
Ethnicity	9i2E	Gypsy/Romany - ethnic category 2001 census
Ethnicity	9i2F	Polish - ethnic category 2001 census
Ethnicity	9i2J	Kosovan - ethnic category 2001 census

Ethnicity	9i2K	Albanian - ethnic category 2001 census
Ethnicity	9i2L	Bosnian - ethnic category 2001 census
Ethnicity	9i2M	Croatian - ethnic category 2001 census
Ethnicity	9i2N	Serbian - ethnic category 2001 census
Ethnicity	9i2P	Other republics former Yugoslavia - ethnic categ 2001 census
Ethnicity	9i2Q	Mixed Irish and other White - ethnic category 2001 census
Ethnicity	9i2S	Other mixed White - ethnic category 2001 census
Ethnicity	9i2T	Other White or White unspecified ethnic category 2001 census
Ethnicity	9i3	White and Black Caribbean - ethnic category 2001 census
Ethnicity	9i4	White and Black African - ethnic category 2001 census
Ethnicity	9i5	White and Asian - ethnic category 2001 census
Ethnicity	9i6	Other Mixed background - ethnic category 2001 census
Ethnicity	9i60	Black and Asian - ethnic category 2001 census
Ethnicity	9i61	Black and Chinese - ethnic category 2001 census
Ethnicity	9i62	Black and White - ethnic category 2001 census
Ethnicity	9i63	Chinese and White - ethnic category 2001 census
Ethnicity	9i64	Asian and Chinese - ethnic category 2001 census
Ethnicity	9i65	Other Mixed or Mixed unspecified ethnic category 2001 census
Ethnicity	9i7	Indian or British Indian - ethnic category 2001 census
Ethnicity	9i8	Pakistani or British Pakistani - ethnic category 2001 census
Ethnicity	9iA	Other Asian background - ethnic category 2001 census
Ethnicity	9iA1	Punjabi - ethnic category 2001 census
Ethnicity	9iA2	Kashmiri - ethnic category 2001 census
Ethnicity	9iA3	East African Asian - ethnic category 2001 census
Ethnicity	9iA4	Sri Lankan - ethnic category 2001 census
Ethnicity	9iA5	Tamil - ethnic category 2001 census
Ethnicity	9iA6	Sinhalese - ethnic category 2001 census
Ethnicity	9iA7	Caribbean Asian - ethnic category 2001 census
Ethnicity	9iA8	British Asian - ethnic category 2001 census
Ethnicity	9iA9	Mixed Asian - ethnic category 2001 census
Ethnicity	9iAA	Other Asian or Asian unspecified ethnic category 2001 census
Ethnicity	9iB	Caribbean - ethnic category 2001 census
Ethnicity	9iC	African - ethnic category 2001 census
Ethnicity	9iD	Other Black background - ethnic category 2001 census
Ethnicity	9iD0	Somali - ethnic category 2001 census
Ethnicity	9iD1	Nigerian - ethnic category 2001 census
Ethnicity	9iD2	Black British - ethnic category 2001 census
Ethnicity	9iD3	Mixed Black - ethnic category 2001 census
Ethnicity	9iD4	Other Black or Black unspecified ethnic category 2001 census
Ethnicity	9iE	Chinese - ethnic category 2001 census
Ethnicity	9iF	Other - ethnic category 2001 census
Ethnicity	9iF0	Vietnamese - ethnic category 2001 census
Ethnicity	9iF1	Japanese - ethnic category 2001 census
Ethnicity	9iF2	Filipino - ethnic category 2001 census
Ethnicity	9iF3	Malaysian - ethnic category 2001 census
Ethnicity	9iF4	Buddhist - ethnic category 2001 census
Ethnicity	9iF5	Hindu - ethnic category 2001 census
Ethnicity	9iF6	Jewish - ethnic category 2001 census
Ethnicity	9iF7	Muslim - ethnic category 2001 census
Ethnicity	9iF8	Sikh - ethnic category 2001 census
Ethnicity	9iF9	Arab - ethnic category 2001 census
Ethnicity	9iFA	North African - ethnic category 2001 census
Ethnicity	9iFC	Israeli - ethnic category 2001 census
Ethnicity	9iFD	Iranian - ethnic category 2001 census
Ethnicity	9iFE	Kurdish - ethnic category 2001 census
Ethnicity	9iFF	Moroccan - ethnic category 2001 census
Ethnicity	9iFG	Latin American - ethnic category 2001 census
Ethnicity	9iFH	South and Central American - ethnic category 2001 census
Ethnicity	9iFK	Any other group - ethnic category 2001 census
Ethnicity	9iG	Ethnic category not stated - 2001 census
Ethnicity	9S	Ethnic groups (census)

Ethnicity	9S12	Other white ethnic group
Ethnicity	9S14	Other white British ethnic group
Ethnicity	9SA	Other ethnic non-mixed (NMO)
Ethnicity	9SA1	Brit. ethnic minor. spec.(NMO)
Ethnicity	9SA2	Brit. ethnic minor. unsp (NMO)
Ethnicity	9SAD	Other ethnic NEC (NMO)
Ethnicity	9SD	Ethnic group not given - patient refused
Ethnicity	9SE	Ethnic group not recorded
Ethnicity	9SG	Other black ethnic group
Ethnicity	9SH	Other Asian ethnic group
Ethnicity	9SJ	Other ethnic group
Ethnicity	9SZ	Ethnic groups (census) NOS
Ethnicity	9T	Ethnicity and other related nationality data
Ethnicity	9T1	New Zealand ethnic groups
Ethnicity	9T11	New Zealand European
Ethnicity	9T11-1	Pakeha
Ethnicity	9T12	Other European in New Zealand
Ethnicity	9T13	New Zealand Maori
Ethnicity	9T14	Samoan
Ethnicity	9T15	Cook Island Maori
Ethnicity	9T16	Tongan
Ethnicity	9T17	Niuean
Ethnicity	9T18	Tokelauan
Ethnicity	9T19	Fijian
Ethnicity	9T1A	Other Pacific ethnic group
Ethnicity	9T1B	South East Asian
Ethnicity	9T1C	Chinese
Ethnicity	9T1D	Indian
Ethnicity	9T1E	Other Asian
Ethnicity	9T1Y	Other New Zealand ethnic group
Ethnicity	9T1Z	New Zealand ethnic group NOS
Ethnicity	9T2	Traveller - gypsy
Ethnicity	9T3	Yemeni
Ethnicity	9T4	Romanian
Ethnicity	9T5	Bulgarian
Ethnicity	9T6	Czech
Ethnicity	9T7	Slovak
Ethnicity	9T8	Portuguese
Ethnicity	9T9	Nepali
Ethnicity	EMISNQBR14	Brazilian
Ethnicity	EMISNQPO8	Portuguese

Group Name	Read code	Description
Metastases	1D18	Pain from metastases
Metastases	B153	Secondary malignant neoplasm of liver
Metastases	B56	Secondary and unspecified malignant neoplasm of lymph nodes
Metastases	B56-1	Lymph node metastases
Metastases	B560	Secondary and unspec malig neop lymph nodes head/face/neck
Metastases	B560-99	Secondary nodes - head/neck
Metastases	B5600	Secondary and unspec malig neop of superficial parotid LN
Metastases	B5601	Secondary and unspec malignant neoplasm mastoid lymph nodes
Metastases	B5602	Secondary and unspec malig neop superficial cervical LN
Metastases	B5603	Secondary and unspec malignant neoplasm occipital lymph node
Metastases	B5604	Secondary and unspec malig neop deep parotid lymph nodes
Metastases	B5605	Secondary and unspec malig neop submandibular lymph nodes
Metastases	B5606	Secondary and unspec malig neop of facial lymph nodes
Metastases	B5607	Secondary and unspec malig neop submental lymph nodes
Metastases	B5608	Secondary and unspec malig neop anterior cervical LN
Metastases	B5609	Secondary and unspec malig neop deep cervical LN
Metastases	B560z	Secondary unspec malig neop lymph nodes head/face/neck NOS
Metastases	B561	Secondary and unspec malig neop intrathoracic lymph nodes

Metastases	B561-99	Secondary nodes -intrathoracic
Metastases	B5610	Secondary and unspec malig neop internal mammary lymph nodes
Metastases	B5611	Secondary and unspec malig neop intercostal lymph nodes
Metastases	B5612	Secondary and unspec malig neop diaphragmatic lymph nodes
Metastases	B5613	Secondary and unspec malig neop ant mediastinal lymph nodes
Metastases	B5614	Secondary and unspec malig neop post mediastinal lymph nodes
Metastases	B5615	Secondary and unspec malig neop paratracheal lymph nodes
Metastases	B5616	Secondary and unspec malig neop superfic tracheobronchial LN
Metastases	B5617	Secondary and unspec malig neop inferior tracheobronchial LN
Metastases	B5618	Secondary and unspec malig neop bronchopulmonary lymph nodes
Metastases	B5619	Secondary and unspec malig neop pulmonary lymph nodes
Metastases	B561z	Secondary and unspec malig neop intrathoracic LN NOS
Metastases	B562	Secondary and unspec malig neop intra-abdominal lymph nodes
Metastases	B562-99	Secondary nodes - intra.abdom.
Metastases	B5620	Secondary and unspec malig neop coeliac lymph nodes
Metastases	B5621	Secondary and unspec malig neop superficial mesenteric LN
Metastases	B5622	Secondary and unspec malig neop inferior mesenteric LN
Metastases	B5623	Secondary and unspec malig neop common iliac lymph nodes
Metastases	B5624	Secondary and unspec malig neop external iliac lymph nodes
Metastases	B562z	Secondary and unspec malig neop intra-abdominal LN NOS
Metastases	B563	Secondary and unspec malig neop axilla and upper limb LN
Metastases	B563-99	Secondary nodes - axilla/arm
Metastases	B5630	Secondary and unspec malig neop axillary lymph nodes
Metastases	B5631	Secondary and unspec malig neop supratrochlear lymph nodes
Metastases	B5632	Secondary and unspec malig neop infraclavicular lymph nodes
Metastases	B5633	Secondary and unspec malig neop pectoral lymph nodes
Metastases	B563z	Secondary and unspec malig neop axilla and upper limb LN NOS
Metastases	B564	Secondary and unspec malig neop inguinal and lower limb LN
Metastases	B564-99	Secondary nodes - inguinal/leg
Metastases	B5640	Secondary and unspec malig neop superficial inguinal LN
Metastases	B5641	Secondary and unspec malig neop deep inguinal lymph nodes
Metastases	B5642	Secondary and unspec malig neop popliteal lymph nodes
Metastases	B564z	Secondary and unspec malig neop of inguinal and leg LN NOS
Metastases	B565	Secondary and unspec malig neop intrapelvic lymph nodes
Metastases	B565-99	Secondary nodes - intrapelvic
Metastases	B5650	Secondary and unspec malig neop internal iliac lymph nodes
Metastases	B5651	Secondary and unspec malig neop inferior epigastric LN
Metastases	B5652	Secondary and unspec malig neop circumflex iliac LN
Metastases	B5653	Secondary and unspec malig neop sacral lymph nodes
Metastases	B5654	Secondary and unspec malig neop obturator lymph nodes
Metastases	B565z	Secondary and unspec malig neop intrapelvic LN NOS
Metastases	B56y	Secondary and unspec malig neop lymph nodes multiple sites
Metastases	B56y-99	Secondary nodes - multiple
Metastases	B56z	Secondary and unspec malig neop lymph nodes NOS
Metastases	B56z-99	Secondary nodes NOS
Metastases	B57-1	Metastases of respiratory and/or digestive systems
Metastases	B570	Secondary malignant neoplasm of lung
Metastases	B571	Secondary malignant neoplasm of mediastinum
Metastases	B572	Secondary malignant neoplasm of pleura
Metastases	B573	Secondary malignant neoplasm of other respiratory organs
Metastases	B574	Secondary malignant neoplasm of small intestine and duodenum
Metastases	B5740	Secondary malignant neoplasm of duodenum
Metastases	B5741	Secondary malignant neoplasm of jejunum
Metastases	B5742	Secondary malignant neoplasm of ileum
Metastases	B574z	Secondary malig neop of small intestine or duodenum NOS
Metastases	B575	Secondary malignant neoplasm of large intestine and rectum
Metastases	B5750	Secondary malignant neoplasm of colon
Metastases	B5751	Secondary malignant neoplasm of rectum
Metastases	B575z	Secondary malig neop of large intestine or rectum NOS
Metastases	B5760	Secondary malignant neoplasm of retroperitoneum
Metastases	B5761	Secondary malignant neoplasm of peritoneum

Metastases	B577	Secondary malignant neoplasm of liver
Metastases	B577-1	Liver metastases
Metastases	B57y	Secondary malignant neoplasm of other digestive organ
Metastases	B58	Secondary malignant neoplasm of other specified sites
Metastases	B580	Secondary malignant neoplasm of kidney
Metastases	B5823	Secondary malignant neoplasm of skin of trunk
Metastases	B5826	Secondary malignant neoplasm of skin of breast
Metastases	B583	Secondary malignant neoplasm of brain and spinal cord
Metastases	B583-99	Secondary Ca brain/spinal cord
Metastases	B5830	Secondary malignant neoplasm of brain
Metastases	B5831	Secondary malignant neoplasm of spinal cord
Metastases	B5832	Cerebral metastasis
Metastases	B583z	Secondary malignant neoplasm of brain or spinal cord NOS
Metastases	B584	Secondary malignant neoplasm of other part of nervous system
Metastases	B585	Secondary malignant neoplasm of bone and bone marrow
Metastases	B585-99	Secondary Ca bone/bone marrow
Metastases	B5850	Pathological fracture due to metastatic bone disease
Metastases	B586	Secondary malignant neoplasm of ovary
Metastases	B587	Secondary malignant neoplasm of adrenal gland
Metastases	B58y	Secondary malignant neoplasm of other specified sites
Metastases	B58y0	Secondary malignant neoplasm of breast
Metastases	B58y5	Secondary malignant neoplasm of prostate
Metastases	B58yz	Secondary malignant neoplasm of other specified site NOS
Metastases	B58z	Secondary malignant neoplasm of other specified site NOS
Metastases	B58z-99	Secondary Ca NOS
Metastases	B594	Secondary malignant neoplasm of unknown site
Metastases	ByuC	[X]Malignant neoplasm of ill-defined, secondary and unspeci
Metastases	ByuC3	[X]Secondary malignant neoplasm/oth+unspc respiratory organs
Metastases	ByuC4	[X]Secondary malignant neoplasm/oth+unspcfd digestive organs
Metastases	ByuC7	[X]Secondary malignant neoplasm of other specified sites
Metastases	EMISNQME41	Metastasis stage M0
Metastases	EMISNQME42	Metastasis stage M1
Metastases	EMISNQME43	Metastasis stage M2
Metastases	EMISNQME44	Metastasis stage M3
Metastases	EMISNQME45	Metastasis stage M4
Metastases	EMISNQME46	Metastasis stage M1a
Metastases	EMISNQME47	Metastasis stage M1b
Metastases	EMISNQME48	Metastasis stage M1c

**Group Name**	**Read code**	**Description**
Smoking status	137-1	Smoker - amount smoked
Smoking status	1371	Never smoked tobacco
Smoking status	1371-1	Non-smoker
Smoking status	1372	Trivial smoker - < 1 cig/day
Smoking status	1372-1	Occasional smoker
Smoking status	1373	Light smoker - 1-9 cigs/day
Smoking status	1374	Moderate smoker - 10-19 cigs/d
Smoking status	1375	Heavy smoker - 20-39 cigs/day
Smoking status	1376	Very heavy smoker - 40+cigs/d
Smoking status	1377	Ex-trivial smoker (<1/day)
Smoking status	1378	Ex-light smoker (1-9/day)
Smoking status	1379	Ex-moderate smoker (10-19/day)
Smoking status	137A	Ex-heavy smoker (20-39/day)
Smoking status	137a	Pipe tobacco consumption
Smoking status	137B	Ex-very heavy smoker (40+/day)
Smoking status	137b	Ready to stop smoking
Smoking status	137C	Keeps trying to stop smoking
Smoking status	137c	Thinking about stopping smoking
Smoking status	137D	Admitted tobacco cons untrue ?
Smoking status	137d	Not interested in stopping smoking
Smoking status	137E	Tobacco consumption unknown

Smoking status	137e	Smoking restarted
Smoking status	137F	Ex-smoker - amount unknown
Smoking status	137f	Reason for restarting smoking
Smoking status	137F-99	EX-Smoker NOS
Smoking status	137G	Trying to give up smoking
Smoking status	137g	Cigarette pack-years
Smoking status	137H	Pipe smoker
Smoking status	137h	Minutes from waking to first tobacco consumption
Smoking status	137I	Passive smoker
Smoking status	137i	Ex-tobacco chewer
Smoking status	137I0	Exposed to tobacco smoke at home
Smoking status	137J	Cigar smoker
Smoking status	137j	Ex-cigarette smoker
Smoking status	137K	Stopped smoking
Smoking status	137k	Refusal to give smoking status
Smoking status	137K0	Recently stopped smoking
Smoking status	137L	Current non-smoker
Smoking status	137l	Ex roll-up cigarette smoker
Smoking status	137L-99	Tobacco Consumption Nil
Smoking status	137M	Rolls own cigarettes
Smoking status	137m	Failed attempt to stop smoking
Smoking status	137N	Ex pipe smoker
Smoking status	137n	Total time smoked
Smoking status	137O	Ex cigar smoker
Smoking status	137o	Waterpipe tobacco consumption
Smoking status	137P	Cigarette smoker
Smoking status	137P-1	Smoker
Smoking status	137Q	Smoking started
Smoking status	137Q-1	Smoking restarted
Smoking status	137R	Current smoker
Smoking status	137S	Ex smoker
Smoking status	137T	Date ceased smoking
Smoking status	137U	Not a passive smoker
Smoking status	137V	Smoking reduced
Smoking status	137W	Chews tobacco
Smoking status	137X	Cigarette consumption
Smoking status	137Y	Cigar consumption
Smoking status	137Z	Tobacco consumption NOS
Smoking status	13p	Smoking cessation milestones
Smoking status	13p0	Negotiated date for cessation of smoking
Smoking status	13p1	Smoking status at 4 weeks
Smoking status	13p2	Smoking status between 4 and 52 weeks
Smoking status	13p3	Smoking status at 52 weeks
Smoking status	13p4	Smoking free weeks
Smoking status	13p7	Smoking status at 12 weeks
Smoking status	13WF-1	Smoker in the family
Smoking status	9ko	Current smoker annual review - enhanced services admin
Smoking status	9ko-1	Current smoker annual review
Smoking status	ASDFGNO1	Non Smoker - Nos
Smoking status	EGTON1024	Rolls own cigarettes
Smoking status	EGTON1025	Current Smoker NOS
Smoking status	EGTON1026	Ex-cigar smoker
Smoking status	EGTON1027	Ex- Rolled Tobacco Smoker
Smoking status	EGTON1028	Ex-smoker NOS
Smoking status	EGTON320	Smoking Status
Smoking status	EGTON321	Cigarette smoker
Smoking status	EGTON322	Ex-Cigarette Smoker
Smoking status	EGTON324	Ex-pipe smoker
Smoking status	EGTON326	Current smoker
Smoking status	EGTON327	Past smoker
Smoking status	EGTON328	Date stopped smoking

Smoking status	EGTONGR11	Grade B light smoker (1-10/day)
Smoking status	EGTONGR12	Grade C moderate smoker (11-20/day)
Smoking status	EGTONGR13	Grade D heavy smoker (>20 Day)
Smoking status	EGTONSM2	Smoking Age
Smoking status	EGTONSM3	Smoking Age Started
Smoking status	EGTONSM4	Smoking Age Ceased
Smoking status	EGTONSM6	Smoking clinic
Smoking status	EMISNQEX4	Ex-tobacco chewer
Smoking status	EMISNQPR396	Primary carer current smoker
Smoking status	EMISNQSM14	Smoking increased
Smoking status	EMISNQSN1	Snuff use
Smoking status	EMISNQTO12	Total time smoked
Smoking status	EMISNQWA11	Waterpipe tobacco consumption
Smoking status	EMISOTS1	Other smoking information
Smoking status	EMISQCO3	Carbon monoxide validation confirms smoker
Smoking status	EMISQDA1	Date of last cigarette
Smoking status	EMISQGR1	Gradual smoking reduction
Smoking status	EMISSMRE1	Smoker (Read codes)
Smoking status	PCSDT1DE9	Dependent smoker
Smoking status	PCSDT1HE1	Heavy smoker review due
